# Roadmap: Why do patients with cancer die?

**DOI:** 10.1038/s41568-024-00708-4

**Published:** 2024-06-19

**Authors:** Adrienne Boire, Katy Burke, Thomas Cox, Theresa Guise, Mariam Jamal-Hanjani, Tobias Janowitz, Rosandra Kaplan, Rebecca Lee, Charles Swanton, Matthew G. Vander Heiden, Erik Sahai

**Affiliations:** 1 Memorial Sloan Kettering Cancer Center, New York, NY, USA; 2 University College London Hospitals NHS Foundation Trust and Central and North West London NHS Foundation Trust Palliative Care Team, London, UK; 3 The Garvan Institute of Medical Research and The Kinghorn Cancer Centre, Darlinghurst, New South Wales, Australia; 4 Department of Endocrine Neoplasia and Hormonal Disorders, The University of Texas MD Anderson Cancer Center, Houston, TX, USA; 5 Cancer Metastasis Laboratory, University College London Cancer Institute, London, UK; 6 Department of Oncology, University College London Hospitals, London, UK; 7 Cancer Research UK Lung Centre of Excellence, University College London Cancer Institute, London, UK; 8 Cold Spring Harbor Laboratory, Cold Spring Harbor, NY, USA; Northwell Health Cancer Institute, New York, NY, USA; 9 Pediatric Oncology Branch, Center for Cancer Research, National Cancer Institute, National Institutes of Health, Bethesda, Maryland, USA; 10 Tumour Cell Biology Laboratory, The Francis Crick Institute, London, UK; 11 University of Manchester, Manchester, UK; 12 Cancer Evolution and Genome Instability Laboratory, The Francis Crick Institute, London, UK; 13 Koch Institute for Integrative Cancer Research, Massachusetts Institute of Technology, Cambridge, MA 02139, USA; 14 Dana-Farber Cancer Institute, Boston, MA 02115, USA

## Abstract

Cancer is a major cause of global mortality, both in affluent countries and increasingly in developing nations. A large number of patients with cancer experience reduced life expectancy and have metastatic disease at time of death. However, the more precise causes of mortality and patient deterioration prior to death remain poorly understood. This scarcity of information, in particular the lack of mechanistic insights, presents a challenge for the development of novel treatment strategies to improve the quality of , and potentially extend life, for patients with late-stage cancer. In addition, earlier deployment of existing strategies to prolong quality of life is highly desirable. In this Roadmap, we review the proximal causes of mortality in patients with cancer, discuss current knowledge about the inter-connections between mechanisms that contribute to mortality, before finally proposing new and improved avenues for data collection, research, and the development of treatment strategies that may improve patients’ quality and duration of life.

## Introduction

The phrase “metastasis accounts for 90% of cancer deaths” is one of the most widely used in cancer research, yet it is overly simplistic, imprecise, and it is difficult to find any primary analysis supporting the statement. Whilst patients with metastatic disease are overwhelmingly more likely to die than patients with non-metastatic cancer(1,2), the determinants of cancer mortality are multifaceted and frequently involve dysfunction of multiple interconnected systems within the body. Understanding the mechanisms underpinning the causes of mortality, and subsequently intervening, has the potential to make cancer a less destructive disease, improving both the quality and length of life for patients with cancer. However, systematic analyses of the acute and root causes of mortality in patients with cancer are scarce, in part because death certificates rarely record enough information to understand the exact reason why the patient died beyond them having a malignancy. Potentially concomitant comorbidities are also not fully recorded, including in most cases the precise event that led to death. Instead, causes of death may be simply listed as “metastatic carcinoma” or “complications of cancer” which give little insight into why a patient actually died. Even in cases where the cause of death may be attributed to a single event, for example a thromboembolism, the underlying cause of that specific event may be complex. Indeed, metastatic cancer leads to perturbed function of multiple organ systems, and importantly, not just the organs to which disease has spread. This is likely due to the exuberant activation of local and systemic inflammatory, tissue repair, and immune-suppressive programmes.

A simple view would be that the death from metastatic disease correlates with the burden of disease. However, evidence suggests that the situation is more complex, with many factors influencing how metastases impact vital functions and ultimately lead to death. Firstly, metastases to different organs will lead to different impacts on overall health. For example, brain metastases can lead to dysfunction of the central nervous system, whereas peritoneal metastases may cause obstruction of the bowel. In addition, the size or extent of metastases may not necessarily correlate with dysfunction of the organ where it is located (3). Second, the production of the molecular mediators of organ dysfunction can vary between metastases and cancers of different origins. Third, individual patient characteristics like age, sex, overall health, pre-existing comorbidities, genetics and socio-economic status vary (4). Together, these factors directly influence the course of, and physiological response to metastatic disease, and can have profound indirect effects by limiting available treatment options and/or the ability of patients to tolerate or complete all intended treatment (5,6). To understand why patients with cancer die, a closer examination of the factors contributing to mortality in patients with, and dissection of the intricate web of causes that shape the frequency and dynamics of death are required. In this Roadmap, we briefly review data considering the immediate causes of mortality, highlight the intricate inter-connections between different aspects of patient deterioration, and conclude with recommendations for future studies of late-stage cancer that may shed new light on this important aspect of cancer biology and medicine. Death may be related to an acute event, but the underlying mechanisms which trigger it may be modifiable or even preventable. In addition, other deaths may be the end stage of a continuum of deterioration, allowing the possibility of targeted intervention to improve quality of life. In addition, it has been noted that early palliative care improves survival (7). Ultimately, increased understanding of the processes occurring in patients with advanced disease should lead to improved strategies to minimise ill-health and suffering at the end of life. Coupled to this, patients and those around them should be enabled to have essential discussions about their wishes and preferences, minimising potentially inappropriate treatments and maximising quality of life (8).

### Acute events leading to mortality

**Figure 1:**
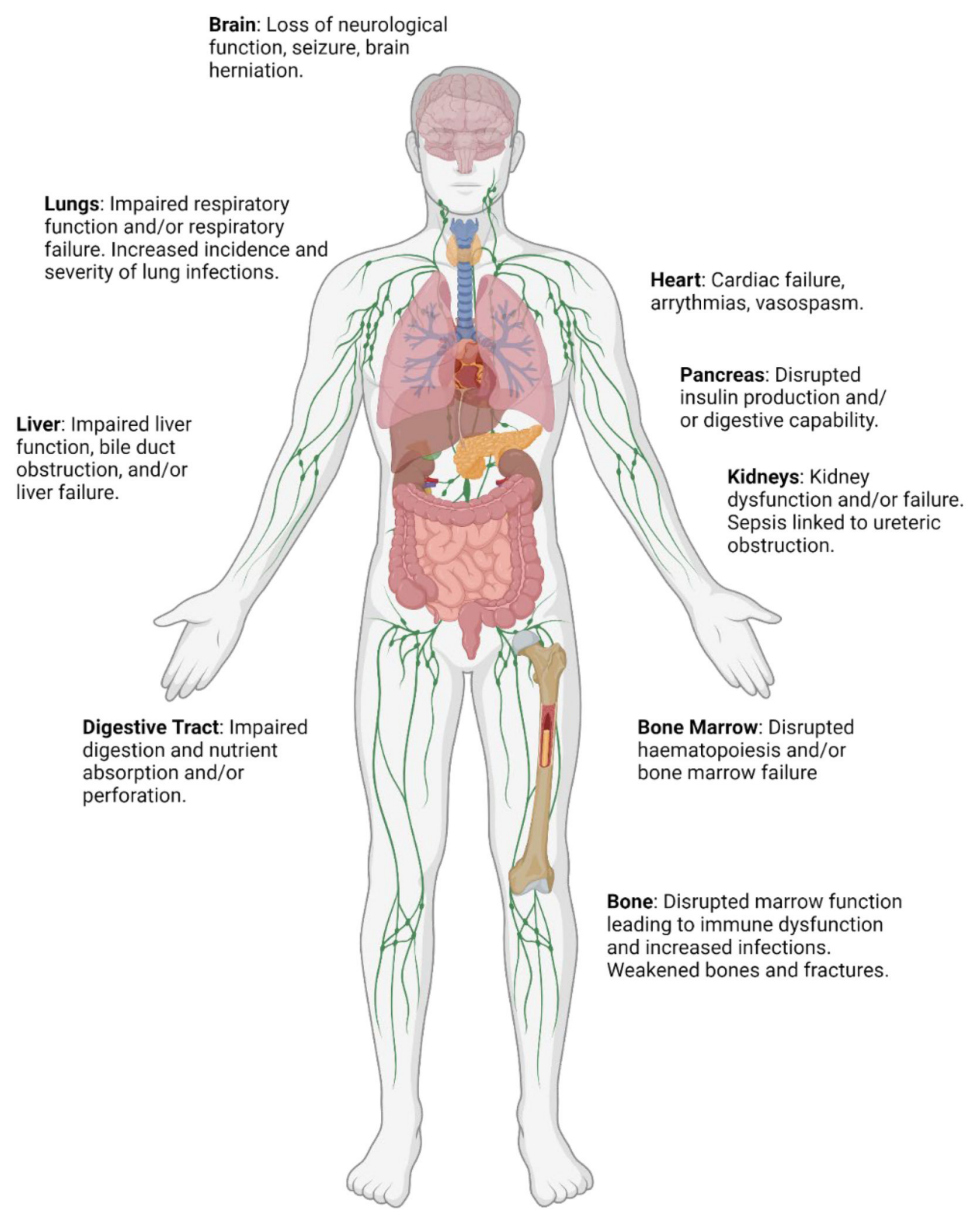
Illustration of the proximal causes of mortality in patients with cancer. Image shows organs that frequently become dysfunctional in late stage cancer patients.

Although some cancers can be considered a chronic disease, with many patients living with their disease for years, the immediate cause of mortality can often be an acute event. Here we briefly summarise common acute events leading to death in patients with cancer (Figure 1). Whilst it is not possible to precisely determine, it is likely that the acute causes discussed below may account for up to half of cancer deaths (9,10). Immediate causes of mortality in other patients are less clear, with a more gradual deterioration typically occurring in vital organ systems.

### Vascular/coagulation/cardiac failure

Patients with cancer are at an elevated risk of thrombo-embolism **[G]**, which may trigger respiratory failure, fatal strokes, heart failure or myocardial infarction (11). In some cases, disseminated intravascular coagulation **[G]** can lead to thrombotic obstruction of small and midsize vessels leading to organ failure (12). Haemorrhagic complications from depletion of platelets, via either immune or non-immune mechanisms PMID: 19466980 PMID: 31205603, and reduced levels of coagulation proteins can also be life-threatening (12). Congestive heart failure **[G]** can also be a proximal cause of mortality, although the underlying causes are complex and include loss of cardiac muscle (associated with cachexia), shifts in intravascular fluid status, and thrombo-embolic events (13). Interestingly, bone metastases are particularly associated with cardiovascular problems, although the underlying mechanism remains unclear (14). Comorbidities affecting the cardiovascular system may make patients more prone to such events. Spatial occlusion or invasion into vessels by cancer metastases can also lead to failure in blood supply or catastrophic haemorrhage (15–18).

### Displacement, functional impairment or obstruction of vital organs

The volume of disease may impair the function of a vital organ. This can be the case with brain metastases and glioblastoma or other primary brain cancers, with either extensive invasion, brain herniation **[G]**, or oedema resulting in midline shift **[G]** or increased intracranial pressure irreversibly compromising brain function (18–20). In addition, patients may develop seizures, which if uncontrolled, can result in death (21,22). However, this does not apply to all brain metastases, with leptomeningeal metastases having minimal impact on intracranial pressure and brain structure; instead, these commonly obstruct cerebrospinal fluid flow and/or affect nerve function resulting in hydrocephalus, deterioration of neurological function, and death (22).

Large lung metastases may impair the essential function of gas exchange. However, patients with miliary-like disease – characterised by nodules too numerous to count – can live with extensive disease in an organ with surprisingly little impact on function until a hard-to-predict tipping point is reached, which is then followed by rapid deterioration (23). As with brain metastases, the volume of disease is often not sufficient to account for organ failure, as even relatively small volume (<100ml lung metastases, compared with 4-5l total lung volume) can be fatal (24). Lung oedema **[G]** related to other pathology such as infection or heart failure can also impair gas exchange causing death, and pleural effusions are an additional common contributor to death. Pleural effusion may be related to presence of disease within the pleura as opposed to total tumour volume (25,26).

Bowel obstruction can be a cause of mortality, particularly in patients with peritoneal disease as found in ovarian, colorectal and gastrointestinal cancers (27). Both liver and kidney failure will also cause death in patients with cancer. Reasons for the failure of these organs include obstruction of the bile duct or ureters by metastases, therapy-induced toxicity leading to compromised normal organ function (discussed below), and reduced tissue perfusion due to hypotension or dehydration (28–31). In addition, sepsis can result from obstruction of the bile ducts or ureters, which occurs unpredictably and often progresses rapidly leading to multiple organ failure and ultimately death.

### Infections

Bacterial infections are the most common infection in patients with cancer, due to impaired immune systems resulting from both the cancer itself as well as certain cancer treatments (discussed in detail in the Iatrogenic effects section), which induce myelosuppression and leukopenia. Patients with cancer can have an elevated risk of opportunistic viral, fungal and protozoal infections, which would typically be considered mild in healthy individuals, but which can cause serious life-threatening complications in those with cancer. Pneumonia and other lung infections leading to respiratory failure are often listed as causes of mortality in patients with cancer (32,33). One of the most striking recent examples of this is the increased mortality observed in patients with cancer, particularly haematological cancers, who succumbed to COVID-19 more than the general population (33,34).

### Paraneoplastic syndromes

Paraneoplastic syndromes are a group of rare disorders that can occasionally cause irreversible damage to critical organs and death. They are most associated with lung, breast, ovarian, and lymphatic cancers, causing tissue or organ dysfunction at sites distinct from the location of the tumour. A variety of mechanisms underpin paraneoplastic syndromes, including the inappropriate production of cytokines, hormones, and antibodies. For example, excess PTHRP production by tumours can lead to hypercalcemia **[G]**. Inappropriate anti-diuretic hormone production is commonly associated with small cell lung cancer resulting in hyponatraemia and some neuroendocrine pancreatic tumours (insulinomas) secrete large amounts of insulin (35–38). Tumours can also trigger the aberrant production of autoantibodies leading to Lambert-Eaton Myasthenic Syndrome, N-methyl-D-aspartate receptor (anti-NMDAR) encephalitis [**G]**, and Myasthenia Gravis (39). Whilst treatment can usually manage the symptoms, however in a subset of cases the syndromes cannot be controlled and are fatal (40).

### Therapy-induced toxicity

Although therapies are developed and administered with the intent of primarily targeting the tumour, almost all have some detrimental impact on normal tissue function. In some cases, the unintended consequences of therapy can be life-threatening. Auto-immune reactions resulting from targeting immune checkpoints can have fatal consequences, including myocarditis and encephalitis (41–43). Death can result from acute neutropenic sepsis related to chemotherapy (44). Depletion of platelets as a result of therapy can lead to fatal bleeding (45). Arrhythmias, cardiomyopathy and coronary vasospasm **[G]** are also a cause of death related to some anti-cancer treatments such as 5-flurouracil and capecitabine (46–48). The long-term detrimental effects of some therapies are discussed in detail in the section on Iatrogenic effects.

### Underlying causes

Determination of the proximal cause of mortality prompts further questions around the underlying factors giving rise to lethal pathology, and ultimately how metastatic cancer triggers or accelerates those factors. In this section, we consider how chronic disruption of three major physiological/organ systems are perturbed in patients with cancer and how these might contribute to mortality.

### The immune and haematopoietic system

In patients with cancer, the immune system becomes progressively less able to mount effective responses to infectious challenge, a phenomenon often generically termed "immune exhaustion" (this usage is distinct from the more specific usage of immune exhaustion as a failure of tumour-reactive T-cells to function). As a result, patients with metastatic disease have increased susceptibility to a wide range of infections, and typically suffer more severe consequences than would otherwise be observed in healthy individuals (49). Multiple mechanisms contribute to the reduced capability of the immune system to respond to infection. The presence of cancer cells in diverse organs triggers similar cellular and molecular events to wound responses (50). The production of cytokines including IL6, G-CSF, and GM-CSF, both by tumour cells and other tumour microenvironment (TME) cells, perturbs haematopoiesis leading to altered profiles of leukocytes(51). While in the short term, this may have limited consequences on the body’s ability to respond to other challenges, prolonged disruption to haematopoiesis can strain the ability of haematopoietic stem cells (HSCs) to generate sufficient cells of the right type to cope with infections, with increased myeloid to lymphoid cell ratios. Clonal haematopoiesis **[G]** can be increased in patients with cancer, with myeloid skewing of immune cells and overall myeloid mediated immune suppression and diminished naïve T cell reservoirs (51). Reduced production of platelets and altered iron metabolism leading to compromised oxygen carrying by red blood cells is also observed in many patients (52). Other problems, such as immunoparesis **[G]** can arise, with a high frequency observed in multiple myeloma patients (53). Once again, comorbidities leading to either immune suppression or auto-immunity can intersect with the detrimental effects of cancer on the immune system. T-cell responses to infection are impaired in the presence of cancer with decreased proliferation and expression of granzyme B typically observed (54). The chronic stimulation of T-cells with neoantigens arising from ongoing mutational processes may also contribute to their weakened functionality. Moreover, immune surveillance of tumours inevitably selects for the production of immune suppressive factors by cancer cells that further compound the issue (55).

Other consequences of cancer result can indirectly result in increased likelihood of infection. For example, vessel obstruction from cancer results in decreased flow of fluids such as bile, urine and lymph, creating environments in which bacteria can thrive (56). Blockage of the bronchial tree can lead to pneumonia (57). The invasive phenotype of cancer can result in fistula **[G]** formation (e.g. recto-vaginal in colorectal cancer) which enables bacteria to invade (58) from one body cavity to the next facilitating spread and subsequent systemic spread leading to sepsis. Furthermore, patients are often rendered bedbound or have limited mobility as cancer progresses, resulting in increased chance of infections through decreased respiratory ventilation and atelectasis **[G]**, as well as pressure sores and oedema (59).

Disruption to haematopoiesis can also contribute to defects in coagulation and haemostasis. Elevated platelet numbers, termed thrombocytosis is found in cancer patients and correlated with higher mortality. The altered inflammatory cytokine milieu caused by the tumour may promote megakaryopoiesis, potentially through increasing Thrombopoietin (TPO) production by the liver, and leading to higher platelet numbers. The risk of clotting can be further increased by the production of tissue factor **[G]**, which is responsible for initiating the clotting cascade, by tumour cells (60). These mechanisms increase the likelihood of fatal thromboembolisms (60).

Iatrogenic effects **[G]** also play a role in the reduced immune function in patients with cancer. Cytotoxic therapies interfere with the proliferation and division of haematopoietic stem cells and can leave the immune system unable to mount effective responses to pathogens, leading to mortality (61). In severe cases, pancytopenia results, marked by a significant decrease in all three major blood cell lineages (red cells, white cells and platelets) (62). This can lead to severe anaemia, increased infection susceptibility, and increased likelihood of bleeding (44,63,64). In other cases, more limited subsets of haematopoietic cells are affected. Thrombocytopenia – low platelet levels – leads to hypo-coagulation and elevates the likelihood of haemorrhage (63). Thus, during cancer development and treatment, haemostasis mechanisms may be either augmented or attenuated, and in both cases the end result is less predictable and well-controlled coagulation. Neutropenia – low neutrophil levels – renders patients less able to fight infection and contributes to cancer mortality from infections that in many cases are thought to arise from resident mucosal flora (65). Treatments, including chemotherapy and radiotherapy, often result in the breakdown of mucosal barriers (e.g. oral mucositis) resulting in higher numbers of infections from pathogens which normally reside on these surfaces (66). In addition, corticosteroids, which are often given to alleviate symptoms or manage toxicity, can also add to suppression of immune response and compound the risk of infections in patients (67). Clonal haematopoiesis, which is already more frequent in cancer patients, can be further increased by chemotherapy (68). More generally, cancer therapies can increase aging-associated processes and reduce organ function (69). The wide-spread use of corticosteroids, used to counteract some of the side-effects of therapy and to reduce the symptoms of cancer, further suppresses the immune system. Opioid pain relief administered to those with late-stage disease can also suppress the function of various bodily systems (70). Finally, infections can arise due to the insertion of drains and stents, or central venous catheters (CVC, also known as lines) for delivery of therapies. Infections from lines is estimated to be around 0.5–10 per 1,000 CVC-days (71,72).

Immunotherapies present a different set of immune complications from conventional therapies. These primarily relate to over-activation of the immune system leading to auto-immunity and, in some cases, cytokine storms that are treated with anti-cytokine therapies such as tocilizumab, anakinra and ruxilitinib, all of which can further suppress the immune response (73). However, deaths attributable to autoimmune side effects of checkpoint inhibitors are rare (approximately 1%) especially if toxicity is managed promptly (74,75). Colitis is a frequent problem, with disruption to colonic barrier function leading to increased susceptibility to perforation, which can be life threatening. In addition, Guillain-Barré syndrome **[G]**, hepatitis, and myocarditis are also causes of checkpoint inhibitor-related deaths (76–78). Once again, high dose corticosteroids are the main first line treatment to manage autoimmune side-effects in patients receiving immunotherapy. A subset of patients experience hyperprogressive disease **[G]** following immunotherapy, the reasons for this are still being delineated but there is likely a role for innate lymphoid cells releasing pro-growth cytokines (79). Cell-based immunotherapies can also lead to disrupted bone marrow function and subsequent myelosuppression (80).

### The nervous system

The brain serves as a central nexus, orchestrating all vital functions. It is the hub of thought processes, emotions, and sensory perception, and regulates, directly or indirectly, everything from heartbeat and breathing, to appetite. In addition to physical disruption of brain structure and intracranial pressure (discussed in the section on immediate causes of mortality) (81), brain metastases impact the nervous system in multiple ways. Tumours in the brain or its surrounding tissues can significantly impair neural connections, leading to cognitive deficits, motor/sensory dysfunction, and even personality changes (81–83). Interactions between brain metastases and neurons lead to changes in cortical function (84–86). Even in regions of the brain without overt metastases, neuro-excitability can be increased, leading to changes in cognition, alertness, and mood (87). Tumours can slow the posterior dominant rhythm, leading to reduced alertness, loss of working memory and deterioration of quality of life (88). Circadian rhythms are also impacted, leading to problems in memory and sleep, which is vital for the body’s repair processes that are essential for overall health and functioning (89). Ultimately, many of these changes are not sustainable long-term. How these changes may lead to death is unclear, but it may follow similar trajectories to those in dementia patients.

Brain function can also be disrupted in patients without brain metastases, with autonomic nervous system dysfunction often reported (90). Intriguingly, anhedonia – a lack of ability to experience pleasure – occurs in many patients (91). The mechanistic causes of this are unclear, but it is not restricted to patients with brain metastasis suggesting that circulating systemic factors may play a role. The wider effects of metastatic cancer on patient’s mental wellbeing are discussed in Text Box 1. However, beyond an effect on well-being, the disruption of brain function can contribute to anorexia, and reduced nutrition can influence many other physiological and pathophysiological processes (92,93).

The role of the peripheral nervous system **[G]** in cancer-related death is not well described. While the burgeoning field of cancer neuroscience provides evidence that the efferent system can support local and metastatic tumor growth (94–96), at this time, it is unclear if the reverse is also true. There is clear evidence of autonomic nervous system dysfunction in patients with cancer (90), raising the possibility that cancer-mediated interruption of afferent impulses might impact overall survival. Further studies are needed to explore this possibility.

### Metabolism and cachexia – catabolic effects of cancer

The presence of metastases presents altered energetic and anabolic demands on the body, leading to detrimental imbalances in metabolism (108). Progressive and involuntary loss of body weight – termed cachexia – is a widespread multiorgan phenomenon commonly seen in patients with metastatic cancer (108–110). This complex syndrome is characterized by a net negative energy balance, driven by the combination of increased energy expenditure and catabolism, with reduced appetite and caloric intake. Persistent decrease in nutrient intake is a key component across patients with many different cancers, leading to breakdown of host tissues, with loss of adipose tissue and muscle mass varying between patients and among different cancers. However, the contribution of increased energy expenditure (as a result of tumour burden) is less clear. Sarcopenia **[G]** may be particularly prominent in some patients, possibly representing an independent pathology from other more global tissue wasting phenotypes, and in extreme cases, loss of cardiac or intercostal muscle mass can be fatal due to insufficient cardiac and/or respiratory function (111,112). These events have also been observed in the context of extreme starvation in patients with non-cancer conditions; for example, anorexia nervosa, where cardiac dysfunction, in particular fatal bradycardia and sinus pauses, can cause pulseless electrical activity and death (113,114). Electrolyte disturbances and hypoglycaemia that are often observed in cases of severe malnutrition may exacerbate the risk of such arrhythmias (113). Cachexia also has effects on other organs, including the brain and immune system. Compromised immune function is a major consequence of starvation-induced tissue wasting, and suggests that altered systemic metabolism leading to, or associated with cachexia, may be a contributor to the immune dysfunction present in some patients with cancer (115). Conversely, several studies have shown that both the brain and immune system can contribute to cachexia (109,110).

Cachexia is multifactorial and has many potential causes. In some limited cases, tumour metabolism leads to systemic changes that increase energy usage. For example, high levels of lactate secretion by tumours can trigger the liver to convert lactate back to glucose, which requires energy input – termed the Cori cycle (116). Such cycles can increase metabolic demand on the liver leading to further perturbation of liver function. However, cachexia does not correlate with disease volume in many cancer types (117). Thus, it is hard to reconcile a model in which the energetic and catabolic demands of the volume of disease are the main trigger for cachexia. Numerous studies have begun to reveal the possible molecular underpinnings of cachexia in some cancer types. Disruption of signalling by TGFβ and related ligands is a recurring theme (118–120). For example, circulating GDF15, a highly conserved member of the TGFβ family, is a known mediator of anorexia and weight loss, and increased circulating levels in patients with lung cancer have been shown to correlate with cachexia development (121). TGFβ itself can also promote muscle loss via the induction of myostatin (122). Induction of signalling by activin – another TGFβ-family ligand – can also have similar effects on muscle mass (123,124). Furthermore, modulation of RyR1 downstream of TGFβ can perturb sarcomere organisation and thereby lead to muscle weakness (125). As such, pre-clinical studies have demonstrated the potential utility of TGFβ blockade in preventing cachexia (126).

Elevated levels of cytokines, including TNFα, IL1, and IL6, can also play roles in cachexia (127–129). TNFα induces multiple aspects of cachexia (130). Muscle wasting is promoted through increased TNFα and NFκB-dependent ubiquitin-mediated proteolysis of muscle protein (131,132). IL6 triggers muscle loss through a similar mechanism. Lipid metabolism is impacted by TNFα reducing the expression of lipoprotein lipase and free fatty acid transporters, thereby reducing the accumulation of fat (133). TNFα can also reduce appetite through the production of corticotropin-releasing hormone **[G]** (CRH). IL1, which triggers similar proximal changes in cell signalling to TNFα, can activate many of the same processes (133). It is also interesting to note that TGFβ, IL1, and IL6 are associated with programmes in cancer cells that drive metastasis, which could potentially explain why metastatic disease is linked to cachexia more strongly than the presence of primary disease alone.

### Whole body dysfunction

Although consideration of different organ systems is useful for highlighting some of the key events contributing to cancer mortality, the inter-connected nature of body systems and the pleiotropic characteristics of the molecular mediators at play mean that ultimately it is essential to consider whole body dysfunction when thinking about causes of cancer mortality. Furthermore, such analyses may explain cancer deaths without an acute proximal cause. As discussed above, cytokines with potent effects on the immune system, as well as effects on appetite, can be contributors to cachexia. Therefore, it is unsurprising that tumours impact both immune and metabolic function. The immune and nervous systems are highly sensitive to metabolite availability; for example, the brain has a high demand for glucose (115,134). Several factors, including lactic acid production and kidney dysfunction can lead to life-threatening systemic acidosis in patients with cancer, particularly haematological malignancies with high cell turnover (135). These can be further exacerbated upon initiation of cytotoxic therapy resulting in tumour lysis syndrome which can be fatal (136). Consequently, metabolic perturbations and cachexia impact these systems. Over time, the cumulative stress of metabolic alterations caused by metastases, chronic changes in the level of cytokines, constant generation of tumour (neo)-antigens, aggressive therapies, and incidental infections lead to exhaustion of the adaptive immune system and hamper the regenerative capacity of many organ systems with debilitating effects (14). This multi-faceted burden can ultimately trigger a body-wide shut-down leading to death.

### Are cancer mortality causes cancer-specific?

Although a subset of mortality causes are cancer-specific, such as metastatic invasion compromising specific organ function, the progressive and inter-connected deterioration of multiple organ systems likely underlies many cancer deaths. This may be further influenced by interaction with other co-morbidities. Of note, similar progressive deterioration is sometimes observed in the context of chronic infection and inflammation, with both cachexia and immune exhaustion being associated with diseases such as tuberculosis (TB) and Human Immunodeficiency Virus (HIV) infection (137–139). This raises the question of whether the causes of death in patients with cancer are specific to cancer, or whether cancer (or any other chronic disease) is simply an accelerant of aging processes occurring in healthy individuals. This hypothesis has practical implications because, if proven, it would suggest that lessons and approaches from other disease contexts could be readily transferable to patients with metastatic cancer. For example, the targeting or modulation of senescent cells is an active area of anti-aging research and numerous pre-clinical studies have indicated that similar strategies can attenuate the systemic effects of cancer (140–142).

### Recommendations

The goal of this Roadmap is to propose ways to improve our understanding of why patients with cancer die and thereby develop better strategies to ameliorate symptoms and prolong life with good quality in cancer patients. To this end, we propose that the following steps would be useful.

### Improved records and reporting

It is notable how infrequent systematic reviews of the precise causes of cancer mortality are. This gap in knowledge, and recognition that this is often simply not known, is a major hindrance to learning and progress. Although improved accuracy of reporting on death certificates would be desirable, it would require a shift in longstanding clinical habits, and may not be easily achievable in healthcare systems under strain. Palliative care primarily focusses on symptom control for patients whilst balancing the potential benefits and burdens of additional diagnosis. Nevertheless, to address the gaps in our knowledge, it would be desirable to fund and establish prospective studies that continue active monitoring of patients as they transition from active disease treatment to palliative care. If possible, monitoring should be non-invasive to not compromise patient comfort at the end of life. The great advances being made in patient monitoring with wearable technologies **[G]** might facilitate this, and could be used for earlier detection of infections enabling quicker intervention. Caregiver involvement in reporting of symptoms may also play a role. Patient/public involvement in this type of research will be critical. In addition, consent to obtain more detailed information from the community/palliative care teams on the contributing factors to death would provide further insight. In addition to information gathered prior to death, research autopsies have the potential to shed further light on the aetiology of death, such as thromboembolic events that may not have been detected in the absence of symptoms or diagnostic testing – discussed in Box 2. Furthermore, the availability of post-mortem samples can aid research into the biological underpinnings of metastases and processes leading to death. The greatest amount of information would be gained from cohorts additionally enrolled into warm autopsy programmes (see Text Box).

### More detailed observational clinical studies

Disease burden is not well correlated with survival; however, we propose that the accurate identification of prognostic factors correlating with survival should provide important insights into what ultimately precipitates mortality. As the cost of both targeted and non-targeted analysis of proteins and metabolites decreases, it should also become more feasible to explore molecular predictors of survival. Once identified, such factors could then be monitored in a targeted way prospectively with the potential to intervene upon where possible. In this setting, both the tumour and patient trajectory would receive precision tailored treatments, the impact of which would need to be studied in randomised controlled trials. Even in the context of early phase trials, additional data could be obtained about patient symptoms in addition to safety considerations and tumour burden. Clinical imaging could also be exploited. Many patients receive CT and PET scans and these contain abundant information about the burden and location of metastases and offer the opportunity to study changes in extent of adipose and muscle tissue and therefore body composition in relation to cachexia. Machine learning and artificial intelligence can be capitalised on to accurately measure these parameters, meaning that what would have previously been prohibitive due to the hours of radiologist time required is now feasible (147,148). In addition to the analysis of scans, the application of machine learning approaches to metabolite, cytokine, immune cell, and wearable technology-derived multi-modal, and multidimensional data may also uncover previously unknown parameters that correlate with mortality (149). As outlined in Text Box 1, incorporating psychosocial metrics into the study of late-stage cancer could also enable improvements in patients’ mental well-being.

### Increasing the relevance of model systems

Pre-clinical models will also have a place in determining the linkage between events found to precede death and cause of death; however, there should be an emphasis on reverse translation of questions from human studies to pre-clinical models. By way of example, this could involve modelling how metastases impinge on the body’s ability to respond to infection by challenging metastatic models with a pathogen. Animal ethics and husbandry considerations mean that mice are housed in controlled environments where exposure to pathogens is rare, and the types of pathogen exposure very narrow, so this type of information is currently lacking. To be optimally informative, practical and ethical complications around studying end-of-life physiology seen in patients need to be considered. Most models are chosen for their rapid progression, often with less than a month between primary or metastatic tumour seeding and death. These are not optimal for studying longer timescale chronic changes in patients. The development of slower progressing models, implementation of multiple lines of treatment and mimicking presence of other co-morbidities should enable models to more accurately recapitulate observations made in patients. Furthermore, most pre-clinical cancer research currently uses young mice that fail to accurately mirror the interplay between aging and cancer seen in humans. Researchers need to recognise the importance of and adopt more age-appropriate mouse models to better understand cancer mortality. In addition, most studies focus solely on tumour burden (which may only be possible at the point of death rather than dynamically) or tumour size as a marker of disease due to the technical challenges of accurately quantifying organ impairment. Furthermore, when survival is reported in mouse studies it is often animal care facility driven ethically humane end-points that mandate euthanasia as the cause of death. Tumour volume response and progression are poor surrogates of mortality in patients (150), therefore better modelling of other metrics of tumour activity and impact on the body system may lead to better drug devlopment. While minimizing and alleviating suffering in experimental animals is critical, ethical considerations limit the ability to study mortality in mice. Thus, an expanded repertoire of analysis would help to understand how metastases impact specific systems and events, including the haematopoietic and nervous systems, as well as whole-body physiology and metabolism. Analysis of small volumes of blood can provide data on metabolites and cytokines, as well as complete blood counts (red blood cells/white blood cells/ Ppatelets) while increasingly sophisticated and automated technology is available to monitor mouse behaviour. It is worth noting that weight loss is frequently used as a humane end point, which indicates that many cancer models trigger cachexia and that with appropriate measurements there is an opportunity to learn more about this phenomenon in existing models. We advocate more detailed reporting of why mice were culled in experimental studies – e.g. tumour volume, weight loss, laboured breathing, complete blood cell counts and blood chemistry.

### Clinical trials

The types of analyses detailed above will provide correlation between different factors and mortality, but not causative linkage. Ultimately, this information depends on testing in the context of clinical trials. Many of the mediators of immune dysfunction and cachexia can now be targeted with function blocking antibodies or forms of receptor traps, and are being actively explored in clinical trials. Several of these interventions were originally developed for chronic inflammatory conditions, which further highlights links between cancer and inflammation. The use of appropriately chosen secondary end-points would provide an opportunity for testing whether correlative associations have a causal basis. In addition, many cancer drug trials stop providing an intervention at the point where a cancer progresses. The mechanisms behind cancer cachexia suggest that trials should be adapted to additionally consider clinical benefit in terms of weight/muscle loss/other specific determinants of efficacy, rather than to solely monitor cancer progression.

## Concluding remarks

While efforts at cancer prevention and the development of curative treatment rightly receive considerable attention, we argue that understanding the precise events leading to cancer mortality should not be overlooked by funding bodies. Understanding the causes of dysfunction across multiple organ systems, may provide novel strategies to manage symptoms of advanced cancer. In addition, better knowledge of the processes leading to death could enable patients and those around them to have essential discussions about their wishes and preferences, minimising potentially inappropriate treatments and maximising quality and enjoyment of life. Further, more precise biomarkers of the likely timing of death may enable patients and their families to better utilise the time that is left. In the longer term, strategies to prevent organ dysfunction should offer considerable benefit to both patients with high tumour burden and those who have low disease burden but die from factors produced by cancer.

## Glossary

Atelectesia: partial collapse or incomplete inflation of the lung
Brain Herniation: Pressure-induced movement of brain tissue
Clonal Haematopoiesis: An aging-associated process in which haematopoiesis becomes dominated by one or a small number of genetically distinct of stem or progenitor cells. Clonal haematopoiesis is linked to an increased risk of haematological malignancies
Congestive Heart Failure: Inability of the heart to pump blood properly
Coronary vasospasm: Constriction of the arteries supply blood to the heart
Corticotropin-Releasing Hormone (CRH): One of the major factors that drives the body’s response to stress
Disseminated intravascular coagulation (DIC): DIC is a rare but serious condition where abnormal blood clotting occurs throughout the body’s blood vessels
Encephalitis: Inflammation of the brain
Fistula: An abnormal connection that forms between two body parts, such as an organ or blood vessel and another often unrelated structure in close proximity
Guillain-Barré syndrome: This syndrome is a rare disorder in which your body’s immune system attacks your nerves that can lead to paralysis
Hypercalcemia: Elevated calcium levels in the blood, often caused by overactive parathyroid glands. Hypercalcemia is linked to kidney stones, weakened bones, altered digestion, and potentially altered cardiac and brain function
Hyperprogressive Disease (HPD): Rapid tumour progression sometimes observed during immune checkpoint inhibitor (ICI) treatment
Iatrogenic effects: Harm caused by cancer treatments, often unavoidable
Immunoparesis: Defined as the marked suppression of polyclonal immunoglobulins in the body
Lung Oedema: Lung or pulmonary oedema is a condition caused by excess fluid in the lungs. This fluid collects in the alveoli compromising function and making it difficult to breathe
Midline Shift: The observation of displacement of brain tissue across the centre line of the brain, suggestive of uneven intracranial pressure
Paraneoplastic syndromes: A group of rare disorders that occur when the immune system reacts to changes in the body triggered by the presence of a neoplasm
Peripheral Nervous System: A dense network of nerves that transmit information from the brain (efferent neurons) to the periphery and conversely transmit information from the periphery to the brain (afferent neurons)
Sarcopenia: Sarcopenia is a condition characterised by loss of skeletal muscle mass and function
Thrombo-embolism: The lodging of a circulating blood clot within a vessel leading to obstruction. Thrombo-embolisms may occur in veins (venous thrombo-embolism) and arteries (arterial thrombo-embolism)
Tissue Factor: a key component of the pathway regulating blood clotting, specifically the receptor and cofactor for factorVII/VIIa
Wearable technologies: Devices worn on the body, typically in the form of accessories or clothing, that incorporate advanced electronics and technology to monitor, track, or enhance various aspects of human life. Examples include smartwatches and fitness trackers

## Figures and Tables

**Figure 2:**
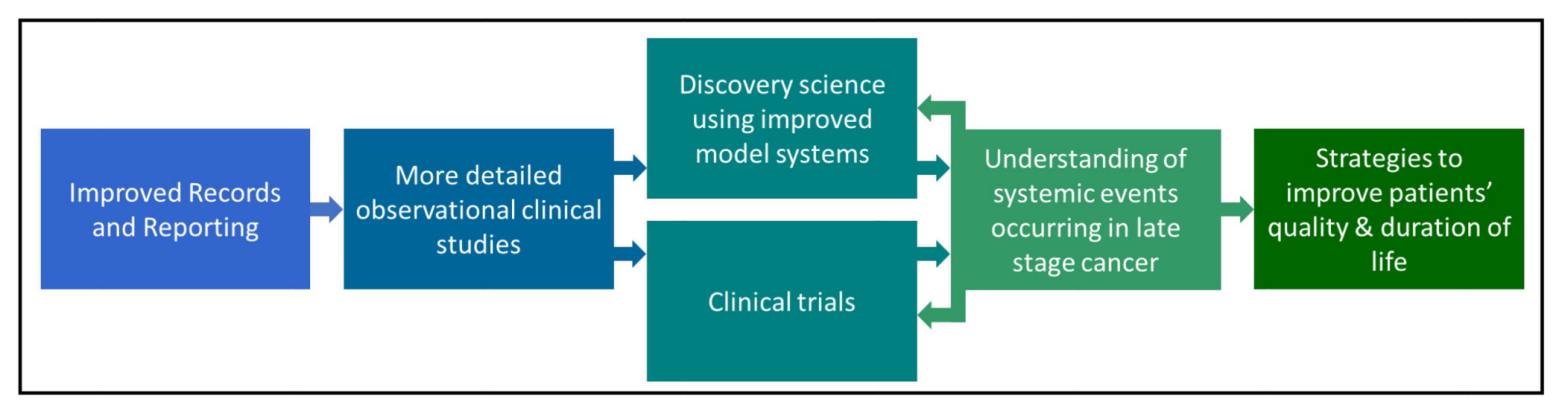
Recommendations for improving understanding of causes of cancer mortality. Scheme shows how recommendations can interlink to provide both improved understanding of the underlying biology and strategies to improve patient’s quality of life.

## TEXT BOX 2 Research Autopsy Programmes and their optimisation

Research autopsies are initiatives that involve the prompt collection of tissues from deceased individuals shortly after death, while tissue morphology is intact, and cells and tissues have not undergone significant post-mortem changes. Research autopsy studies can be labour intensive, and care is required in their logistical planning. The post-mortem interval (PMI) to autopsy can vary depending on the infrastructure available and can have implications for the utility of samples collected after death. For example, shorter PMIs achieved in rapid warm autopsy studies can more effectively facilitate in vitro (e.g. cell line) and in vivo (e.g. organoid and xenograft) models, and can derive better quality RNA (143,144). However, such studies are not easily established in the absence of out-of-hours facilities and expert input. Autopsies performed with longer PMIs, for example up to several days after death, have been shown to have maintained tissue morphology and adequate DNA and RNA to facilitate cellular imaging techniques and genomic sequencing approaches (145,146). Therefore, there is merit and general scientific value with autopsies regardless of the PMI, provided consideration is given to the question being addressed, and the experimental approach.

The most powerful data are obtained from patients already involved in clinical studies prior to death. Information about disease course, longitudinal scans, tissue and blood analysis (cell counts, electrolytes, cytokines, metabolites, and possibly ctDNA) greatly enhances what can be learnt from post-mortem tissues. Sensitivity is required to align the desire to acquire data with the wishes of the patients and their families, such that ultimately each autopsy has the potential to be meaningful and shed light on the biological processes leading to death.
